# Supplementary material for: Complexome profiling of the Chlamydomonas psb28 mutant reveals TEF5 as an early PSII assembly factor
Source: Plant Cell. 2025 Mar 17;37(6):koaf055. doi: 10.1093/plcell/koaf055 (PMC12164590; doi:10.1093/plcell/koaf055)
Supplement: koaf055_Supplementary_Data [file koaf055_supplementary_data.zip › psb28_interactive.zip!/plots/intensity/qualityPlots/peptides/_Cre12.g501550.t1.2.html]

### Cre12.g501550.t1.2

Trivial name:   
SeqCoverage [median]: 45.10  
Mass: 11.83 kDa  
  
 

| Sequence | MissedCleavage | Mass [Da] | wtIntensity | muIntensity | Score |
| --- | --- | --- | --- | --- | --- |
| AEQAPTEPATPKPEAPSTSGEK | 1 | 2222.07 | 1229.07 | 4456.25 | 80.16 |
| AEQAPTEPATPKPEAPSTSGEKIEVK | 2 | 2691.36 | 4254.63 | 9783.32 | 85.85 |
| GGQMLPPPPEALIP | 0 | 1415.75 | 5564.69 | 0.00 | 121.90 |
| KVDASAPLLQK | 1 | 1168.68 | 29270.57 | 62495.98 | 122.70 |
| VDASAPLLQK | 0 | 1040.59 | 3766.35 | 12164.33 | 123.16 |
|  |  |  | 44085.3 | 88899.9 |  |
